# Waiting for it: Anorexia Risk, Future Orientation, and Intertemporal Discounting

**DOI:** 10.21203/rs.3.rs-4002723/v1

**Published:** 2024-03-27

**Authors:** Isabel Schuman, Jingyi Wang, Ian C. Ballard, Regina C. Lapate

**Affiliations:** 1 Department of Psychological & Brain Sciences, University of California, Santa Barbara; 2 Department of Psychology, University of California, Riverside

## Abstract

Anorexia Nervosa is a severe eating disorder characterized by food restriction in service of a future goal: thinness and weight loss. Prior work suggests abnormal intertemporal decision-making in anorexia, with more farsighted decisions observed in patients with acute anorexia. Prospective future thinking in daily life, or temporal orientation, promotes more farsighted delay discounting. However, whether temporal orientation is altered in anorexia, and underlies reduced delay discounting in this population, remains unclear. Further, because changes in delay discounting could reflect cognitive effects of an acute clinical state, it is important to determine whether reduced delay discounting is observed in subclinical, at-risk samples. We measured delay discounting behavior and temporal orientation in a large sample of never-diagnosed individuals at risk of anorexia nervosa. We found that farsighted delay discounting was associated with elevated risk for anorexia nervosa. Anorexia nervosa risk was also associated with increased future-oriented cognition. Future-oriented cognition mediated the difference in delay-discounting behavior between high and low-risk groups. These results were unrelated to subjective time perception and were independent of mood and anxiety symptomatology. These findings establish future-oriented cognition as a cognitive mechanism underlying altered intertemporal decision-making in individuals at risk of developing anorexia nervosa.

## Introduction

### Overview

The ability to guide behavior according to long-term goals is often adaptive—it is linked to higher academic performance, professional success, and favorable health outcomes ^1,2^. For instance, the propensity to prefer smaller rewards *now* as opposed to larger rewards in the *future*—termed delay discounting—is associated with increased likelihood of substance abuse, gambling, bipolar disorders, and depression (for reviews, see: ^2–4^). A notable exception to this seemingly advantageous decision-making profile is seen in *Anorexia Nervosa*, a severe eating disorder characterized by persistent restrictions in calorie intake in service of a subjectively preferred future goal: thinness and weight loss ^5^, which culminates in extremely low body weight, long-lasting impairments physical and mental health, and one of the highest mortality rate amongst psychiatric conditions ^6,7^. Efficacious future-oriented control of behavior, combined with altered reward processing, may underlie the unique capacity for anorexia patients to self-starve despite the inherently rewarding nature of food ^8–12^*.* Accordingly, delay discounting is *reduced* in acute anorexia compared to healthy controls ^13–17^, which contrasts with findings in nearly all other psychiatric disorders ^2,4^.

However, the cognitive mechanisms underlying the association between anorexia and reduced delay discounting remain unclear. How may information be processed differently in anorexia, such that future rewards are not as sharply discounted as they are for most individuals? A growing body of work on intertemporal discounting suggests that a future-oriented cognitive style—i.e., the tendency to spontaneously consider the future when making decisions—may be linked to less steep discounting of future rewards by facilitating the representation of future decision outcomes via episodic prospection or future “time travel” ^3,18–22^, an idea that has not been tested in individuals with anorexia. In addition, prior work characterizing the relationship between delay discounting and anorexia has primarily uncovered associations when examining *acute* anorexia—whereas individuals who have recovered may show delay discounting behavior that is comparable to healthy controls ^4,23^. Thus, whether *reduced* delay discounting is a vulnerability factor for anorexia—and may constitute a risk cognitive endophenotype—remains unclear. Therefore, the present study sought to (1) extend findings of intertemporal discounting abnormalities in acute anorexia to a never-diagnosed, at risk population for eating disorders/anorexia (henceforth, anorexia risk)^1^ and (2) investigate whether individual differences in temporal orientation—specifically, a future-oriented cognitive style—is associated with anorexia risk and may explain aberrant intertemporal decision making in this population.

### Anorexia & Intertemporal Discounting

Delay discounting paradigms model choice preferences in one’s subjective evaluation of present and future rewards ^2,3,29^. Concretely, volunteers are typically asked to choose between two hypothetical monetary amounts (e.g., $20 now or $30 in one week), which vary in value and temporal availability. By modeling choice behavior across hundreds of trials, a reliable *discounting rate*—reflecting the degree to which an individual discounts the value of a monetary reward as the delay to reward receipt increases —is obtained per individual.

Delay discounting rates are predictive of impatience and impulsivity in across several domains that involve making trade-offs between present and future rewards—such as academic and professional performance, substance use, exercise, and health behaviors ^30–36^. While healthy individuals typically prefer smaller-but-sooner rewards—and discount larger-but-later rewards according to a hyperbolic function ^37^—individuals with acute anorexia often show *lower* discounting rates compared to healthy controls and individuals with diagnosed with other disorders ^13–17,38^; for a review, see ^39^. For instance, Steinglass (2017) found that anorexia patients discounted monetary rewards less steeply than participants with common co-occurring diagnoses like substance abuse disorder and obsessive compulsive disorder, as well as healthy controls ^14^.

However, whether *reduced* delay discounting is a cognitive endophenotype for anorexia—i.e., a risk factor causally contributing to the incidence and/or severity of this disorder—remains unclear. While delay discounting is a promising measure that is sensitive to decision-making abnormalities across several distinct psychiatric conditions ^2,4^—upon weight restoration or recovery from acute anorexia, delay discounting rates have been found to return to those of healthy controls ^15,17,40,41^. Thus, it is possible that reduced delay discounting in anorexia may result from cognitive alterations associated with acute starvation—or, alternatively, that in-patient anorexia interventions may themselves alter (and correct) intertemporal decision-making behaviors. Therefore, sampling delay discounting in individuals who are at-risk for (but not yet diagnosed with) anorexia compared a low-risk sample is required to elucidate whether intertemporal decision making may be a vulnerability factor for anorexia.

### Intertemporal discounting mechanisms: Temporal processing alterations in anorexia?

The basic cognitive and affective mechanisms that may drive aberrant intertemporal decision making in anorexia—and account for reduced delay discounting in this population—remain poorly understood. Prior empirical and theoretical work in healthy samples highlights three component processes that jointly contribute to temporal choice patterns: reward valuation, cognitive control, and prospective future thinking ^2,3,42–45^.

Prospection is the process of imagining possible future episodes and outcomes ^46^ and is thought to play a critical role in future reward evaluation and facilitate cognitive control of behavior toward long-term goals ^47^. The propensity to mentally time travel into future episodes when making decisions (also called *episodic future thinking)* permits distal outcomes to be simulated and experienced more vividly and concretely, and “engenders a greater consideration of the future consequences of the choices one makes in the present” ^3^. Indeed, a growing body of work underscores that the way *time* is represented and incorporated during decision making—including the tendency to consider future consequences during everyday life decisions, the vividness of future imagery, and the accuracy of time estimation—shapes temporal discounting behavior. For instance, context manipulations that promote envisioning temporally distal rewards more concretely reduce delay discounting, presumably by lending them heavier weight when considering intertemporal trade-offs ^48–51^. Consistently, individuals who show a future-oriented thinking style—characterized by the propensity to consider future consequences (vs. considerations about the present or the past) typically show reduced delay discounting (as measured by the Zimbardo Time Perspective Inventory (ZTPI) ^21,52^ and the Consideration of Future Consequences (CFC) ^18–20,22,53^. A prior study of a clinical sample found that individuals with anorexia display increased future-oriented cognition as measured by the ZTPI ^54^. However, whether future-oriented cognition is enhanced in individuals at risk for anorexia— and whether this factor may account for altered delay discounting behavior in this population— remains unknown.

During prospective thinking, the accuracy of temporal (e.g., delay) representations has itself been suggested to modulate intertemporal choice behavior, such that the *over*estimation of time may result in the devaluing of future (vs. immediate) rewards ^45^. Accordingly, overestimation of future durations is associated with steeper delay discounting rates and increased preference for immediate rewards ^44,55^. Relatedly, impulsive individuals typically over-estimate the duration of temporal intervals ^56–58^ (reviewed in ^45^). Although few empirical studies have explored temporal processing alterations in anorexia, a recent study found that adolescents with anorexia underestimated temporal durations compared to healthy controls ^59^, raising the possibility that subjective time perception may be impacted in individuals at risk for this disorder.

### The Current Study

Collectively, the above-reviewed findings in clinical samples raise the possibility that inter-temporal discounting may be altered in individuals at risk for anorexia, putatively due to alterations in future-orienting cognition. First, we aimed to extend the prior literature indicating low intertemporal discounting in individuals with acute anorexia ^13–17^ to a subclinical, never-diagnosed population, which would point to altered intertemporal choice as a potential endophenotypic risk factor for anorexia. Building on prior findings, and in light of the capacity of individuals with anorexia to override present-moment appetitive drives in service of future goals (such as thinness and weight loss), we hypothesized that individuals at higher risk for developing anorexia would exhibit reduced intertemporal discounting [log(*k*)]. Following our pre-registration, we also estimated intertemporal discounting using a newer computational model (Ebert-Prelec) that parses intertemporal discounting into impatience (‘a’) and time sensitivity (‘b’) parameters to examine whether it would provide additional insight into specific decision-making processes may be altered in anorexia (pre-registered exploratory analyses; *Supplementary Results*).

Second, we sought to investigate whether future-oriented thinking is increased in a subclinical anorexia group, and whether reduced intertemporal discounting in this population is linked to individual differences in future-oriented cognition. To that end, we obtained a latent score indexing individual differences in future-oriented cognition using two well-validated self-reported questionnaires (the Zimbardo Time Perspective Inventory (ZPTI) and the Consideration of Future Consequences (CFC) and a temporal orientation task (Preoccupation with future events; PFE). We hypothesized that individuals at higher risk for anorexia would show increased orientation towards the future. Differences in discount rates [log(*k*)] and future orientation by anorexia risk group (low, high) were examined using independent-samples t-tests as the primary analysis following our preregistered analysis plan, given the established clinical significance of the EAT cutoff value adopted here and elsewhere ^24,25^. Following our pre-registration and in alignment with the RDoc framework, we additionally examined linear associations amongst those variables using a continuous approach as additional analysis using Pearson’s correlation coefficients (see *Supplementary Results*).

**Figure 1.**
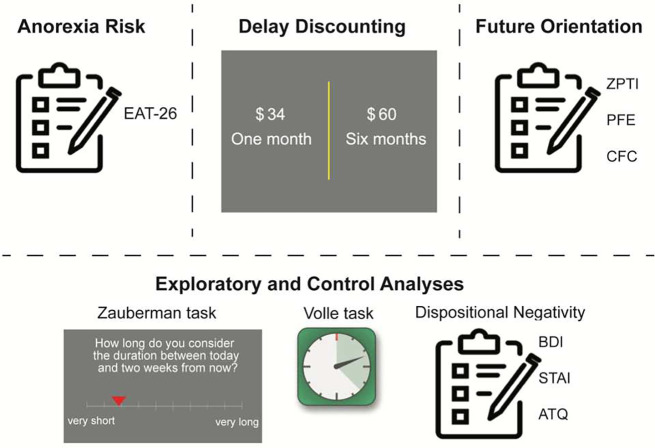
Overview of experimental procedures. The *Eating Attitudes Test* (EAT-26) questionnaire was used as a metric of participants’ anorexia risk. To index intertemporal behavioral choices, participants completed a delay discounting task. Future-oriented cognition was measured using a latent factor derived from the data of 2 questionnaire (ZTPI & CFC) and one task data (PFE). Pre-registered exploratory and control analysis included subjective time measures (the Volle and Zauberman tasks) as well as dispositional negativity, a factor score derived from mood and anxiety questionnaires (BDI, ATQ, and STAI).

In light of prior evidence suggesting that the way time is subjectively experienced can account for variation in inter-temporal choice ^45^, we included tasks assessing subjective temporal processing accuracy in our study, which were examined in pre-registered exploratory analyses. Finally, because anxiety and depression are often comorbid with anorexia, and have been associated with intertemporal choice ^14^, participants completed well-validated mood questionnaires (BDI, STAI, ATQ), which allowed us to examine whether mood and anxiety symptoms accounted for the hypothesized associations between anorexia risk, future-oriented cognition, and intertemporal choice (Figure 1).

## Methods

### Data availability

This study was preregistered on OSF (https://osf.io/h46x2). Any discrepancies between the preregistered plan and reported analyses are described in *Supplementary Materials (see Preregistered vs. reported analysis).* Study materials, including the data, scripts used for running the experiment, and data analysis scripts, can be found on OSF: https://osf.io/3y7dq/.

### Participants

A preregistered sample of *n* = 152 individuals (Age range = 18–51; *M* = 27.33 *SD* = 9.68; n = 109 females, 40 males, 3 non-binary) completed the experiment online in Pavlovia (https:/pavlovia.org) (See sample size justification in the preregistration: https://osf.io/h46x2). Demographic information excluded 2 participants who chose not to provide it. Participants were recruited from UC Santa Barbara using SONA (n = 62) and from the broader community using Prolific (n = 90). Eligible participants were fluent English speakers, with normal or corrected-to-normal vision, and at least 18 years of age. Following our pre-registered data inclusion criteria, n = 16 participants were excluded from delay discounting analysis due to poor compliance and/or poor data quality in the delay discounting task, and n = 3 were excluded from temporal orientation analysis due poor questionnaire data quality (see Data quality exclusion criteria below for details). This resulted on a final sample of n = 136 individuals for analysis of the delay discounting task data (Age range = 18–51; *M* = 27.18 *SD* = 9.46; n= 97 females, 36 males, 3 non-binary; n = 49 of those individuals fell in the high-risk category (Age *M* =30.8, *SD* = 10.16; n = 37 female, 12 male) and n = 87 in the low-risk category (Age *M* = 25.12, *SD* = 8.43; n = 60 female, 27 male)). The final sample for analysis of temporal orientation comprised n = 149 participants (Age range = 18–51; *M* = 27.22; *SD* = 9.66; n= 107 females, 39 males, 3 non-binary; n = 55 of those individuals fell in the high-risk category (Age *M* = 30.2, *SD* = 10.04; n = 42 female, 13 male) and n = 94 in the low-risk category (Age *M* = 25.46, *SD* = 9.03; n = 65 female, 29 male)) (See Data quality exclusion criteria & *Supplementary Table 1* for details). Written informed consent was obtained from all subjects and/or their legal guardian(s). All procedures were approved by the Human Subjects Review Committee at the University of California, Santa Barbara. Participants were compensated for their participation with course credit or payment. all methods were carried out in accordance with relevant guidelines and regulations.

#### Data quality exclusion criteria.

##### Delay discounting task.

Following our pre-registered plan to maximize data quality and verify task compliance in this online study, data quality checks (i.e., catch trials) were included in the delay discounting task, and low-quality data (due to insufficient data and/or unreliable model fits) were excluded from analyses, as follows. We administered randomly placed catch trials that asked participants to choose between a “smaller later” reward and a “larger sooner” reward (e.g., $10 today *vs.* $5 in two weeks; see *Supplementary Table 2*). We excluded participants from analysis of the delay discounting task if they failed a catch trial (e.g., “smaller later” reward), and/or if their model fitting raised a warning due to random responding behavior detected by the hierarchical Bayesian inference model (cf. ^60^). Eight (out of 152) participants failed a catch trial, and 8 (out of the remaining 144) participants’ hierarchical hyperbolic model fits raised a random-responding warning. Thus, a total of n=16/152 participants were excluded due to poor-quality data in the delay discounting task, rendering the final sample n=136 (final demographics are detailed above).

##### Future Orientation questionnaire.

Following our pre-registered plan, participants’ questionnaire data were excluded if they failed to respond to at least 75% of a questionnaire’s items and/or if their score exceeded 3 SD from the mean. Three participants (out of n = 152 eligible participants) were excluded from analysis following this criterion; one participant due to insufficient responses in the Preoccupation with Future Events Questionnaire, one participant due to insufficient responses in the Consideration of future consequences, and one participant due to outlying values in the future-orientation factor scores.

### Materials & Procedures

#### Eating Disorder Risk & Group Assignment

We assessed eating disorder risk using the *Eating Attitudes Test (EAT-26),* a well-validated 26-item questionnaire that assesses an individual’s likelihood of developing an eating disorder ^24^. Scores on the EAT-26 reliably index eating disorder risk and are widely used to identify at-risk individuals ^25^. For each item, participants indicate how often they experience each thought or action, ranging on a 5-point Likert scale from “Never” to “Always”. For items 1–25, “Never, Rarely, Sometimes” are coded as 0, “Often” is coded as 1, “Usually” is coded as 2, and “Always” is coded as 3. For item 26, “Always, Usually, Often” are coded as 0, “Sometimes” is coded as 1, “Rarely” is coded as 2, and “Never” is coded as 3. Scores above 20 indicate that an individual is “high risk” and scores below 20 indicate “low risk” ^24^. Following our pre-registration, participants were assigned to either the “high risk” or “low risk” group based on this criterion.

#### Future Orientation

The following questionnaires were used to derive the latent factor *Future Orientation*, which was used for subsequent analysis: Consideration of Future Consequences Scale ^53^, the Zimbardo Time Perspective Inventory ^52^ and Preoccupation with Future Events ^61^. These questionnaires are described below.

#### Consideration of Future Consequences.

The extent to which participants self-report taking future consequences into consideration during everyday-life decision making was measured using the Consideration of Future Consequences Scale (CFC) ^53^. The CFC is composed of 12 statements and participants are asked to indicate how characteristic of them they find each statement to be, ranging from “extremely uncharacteristic” to “extremely characteristic”. For items 1, 2, 6, 7, and 8, “extremely uncharacteristic” is coded as 1, “somewhat uncharacteristic” is coded as 2, “uncertain” is coded as 3, “somewhat characteristic” is coded as 4 and “extremely characteristic” is coded as 5. For items 3–5 and 9–12 the scores are reversed. Scores on each item were summed to produce a total score ranging from 12–60, with higher scores reflecting greater consideration for future consequences.

#### The Zimbardo Time Perspective Inventory.

Participants’ temporal orientation—i.e., the extent to which they think about future goals (*versus*, for instance, enjoyment of the present-moment) into account when making decisions—was measured using the Zimbardo Time Perspective Inventory (ZTPI) ^52^. The ZTPI is a 56-item questionnaire composed of 5 individual subscales representing different temporal orientation factors, including past-negative, past-positive, present-hedonistic, present-fatalistic, and future. Participants were asked to indicate how characteristic of themselves each statement was, on a five-point scale ranging on a five-point scale ranging from 1 (extremely uncharacteristic) to 5 (extremely characteristic). A higher score represents a greater orientation towards that factor. Average scores were obtained for the future-orientation subscale.

#### Preoccupation with Future Events.

Participants’ preoccupation with future events was assessed using a procedure following Klineberg (1968) ^61^. Participants were asked to write down ten things that they thought about in the prior two weeks. Next, participants examined each thought they wrote down, and indicated whether that thought primarily pertained to the past, present, or future when they were thinking about it. Preoccupation with future events was determined for each participant by calculating a ratio of future thoughts relative to total thoughts listed.

#### Latent Factor Analysis.

A principal component analysis was run to derive a future-orientation latent factor from the above-described task and questionnaires, using singular value decomposition-based principal component analysis with varimax rotation (principal function; Psych R package) ^62^, following our pre-registered data analysis plan. We found that the first component of the three-questionnaire solution accounted 54.6% of the variance of the questionnaire data, with loadings as follows: λ_cfc_ = 0.856; λ_ztpi_future_ = 0.864; λ_PFE_future_ = 0.398. Because λ_PFE_future_ had lower-than-expected loading, we additionally ran a second PCA using the CFC and ZTPI scales only. We found that these two questionnaires explained 78.5% of the variance of the first latent factor, with loadings as follows: λ_cfc_total_ = 0.886; λ_ztpi_future_ = 0.886. Given the improved latent factor solution, we examined whether our results remained consistent when considering the two-questionnaire factor scores; all significant results reported below remained consistent if using the two (vs. pre-registered three-questionnaire-based) factor score. Therefore, we report the results with our pre-registered three-questionnaire latent score analysis. Individual differences in factor loadings were examined in relation to temporal discounting parameters [log(*k*)] and eating disorders risk (EAT scores), as detailed below.

#### Dispositional Negativity

Given the comorbidity of anorexia and mood and anxiety symptoms, participants completed three well validated trait mood and anxiety questionnaires: the *Beck’s Depression Inventory* (BDI)^63^, the *State-Trait Anxiety Inventory* (STAI)^64^, and the *negative affect* (NA) subscale from the short-form of the *Adult Temperament Questionnaire* (ATQ)^65^. We ran a factor analysis (PCA) using those questionnaires (as delineated above) to obtain a latent factor of negative mood and anxiety, which we term *dispositional negativity.* The three questionnaires collectively explained 77.7% of the variance of the dispositional negativity factor, and all had loadings above 0.8 (λ_BDI_ = 0.871; λ_STAI_ = 0.935; λ_NA_ = 0.836).

#### Delay Discounting Task

Intertemporal discounting was assessed by asking participants to make a series of hypothetical binary choices between two monetary rewards that differed in their amount and receipt time. For each trial, individuals chose between a reward that was lower in value but would be received earlier in time (“smaller sooner”), versus a reward that was greater in value but would be delivered after a delay (“larger later”). The temporal discounting task was modeled after McClure et al. (2004) and Decker et al. (2015) ^15,29^. The smaller-sooner rewards ranged from $15-$85. The relative difference in reward magnitudes between smaller-sooner and larger-later rewards (*r*_*larger-later*_*/r*_*smaller-sooner*_ −1) ranged from 2% to 100%. Thus, larger-later rewards varied between $16-$170. This experimental choice set follows prior work as well as internal piloting, and was carefully selected to produce enough response variability required to estimate individual discount rates. Delay times until receipt of the smaller-sooner reward were either *today*, *2 weeks*, or *1 month*, which were randomly selected. Delay times to the larger-later reward ranged from 1 week to six months in the future and were always greater than the delay times to the smaller-sooner reward (see *Supplementary Table 3*). The screen position of smaller-sooner and larger-later reward options (i.e., to the left vs. right side of the screen) was counterbalanced across trials. Choice trials were self-paced. Responses selected their responses using a button press (“left” vs. “right” key). Each choice was followed by a 1.5 s inter-trial interval. The experiment contained n=144 task trials (plus an additional n=2 catch trials for participants run using the SONA system, and n=10 catch trials for participants run using Prolific) and took approximately 30 min to complete. Participants were offered three breaks during the task. Participants were instructed that there were no right or wrong answers, and asked to indicate which choice they would genuinely prefer to receive. Note that rewards used in this study were hypothetical rewards, which are known to produce behavioral choices that are highly correlated with behavioral choices for real rewards ^66,67^.

#### Temporal Accuracy Tasks

We examined participants’ temporal processing accuracy using two behavioral tasks: the Volle Time Estimation Task ^68^ and the Zauberman Future Time Estimation Task ^55^. Because these tasks had not been previously used in a study of an eating disorder population to the best of our knowledge, these analyses were pre-registered as exploratory.

##### Volle Time Estimation Task.

In this silent counting task (adapted from ^68^), participants were asked to estimate the passage of time until a specific target time interval (ranging from 20 to 50 seconds) was reached. Sequential numbers (ranging from 1 to 10) were flashed on the computer screen (0.1 s/each) at a given frequency—every 1s or every 2s—with the target time interval displayed on the lower right corner of the screen. Flashing numbers disappeared after 10 digits. Participants were asked to continue to silently count according to the flashing numbers’ frequency until they reached the target time interval. For the first 4 trials, flashing numerals were displayed every 1s (1 Hz), with the following target time intervals: 20, 30, 40, and 50. For the last 4 trials, numerals flashed every 2s (0.5 Hz), with the following target time intervals: 15, 20, 25, and 30. Time estimation accuracy was obtained by calculating the difference between participants’ time duration estimates relative to the actual (objective) target interval time for that trial.

##### Zauberman Future Time Estimation Task.

In this task adapted from Zauberman et al., (2009)^55^, participants were asked to consider various time intervals from the present day—ranging from 1 week to 10 years into the future—and to indicate how long they perceived each time interval to be. To do so, they placed a marker using a mouse on a slider that was anchored on the labels “very short” (left) and “very long” (right). The time intervals to be estimated were presented sequentially, with the shortest one (i.e. 1 week from today) asked on the first trial. To facilitate participants’ usage of the scale, they were told at the beginning of the task that the largest time interval they would be asked about would be 10 years from the present date. We calculated the growth ratio (i.e., subjective/objective passage of time) by assessing individual’s prospective time estimation for each trial. Using “1 week” as the anchor, we computed objective growth as relative time differences between the current trial vs. 1 week [i.e., (Time_trial_ − Time_anchor_)/Time_anchor_]. Accordingly, subjective growth was computed as the relative slider response differences between the current trial and 1 week [i.e., (Slider response_trial_ – Slider response_anchor_)/Slider response_anchor_]. Growth ratio was computed as the subjective growth divided by objective growth, such that a growth ratio closer to 1 indicated a more accurate prospective time estimation.

#### Data processing and analysis

##### Delay Discounting Task.

As the primary analysis of the delay discounting task, we fit participants’ choice data using a hierarchical Bayesian model implemented in Vincent’s delay discounting toolbox 1.7.1 (https://drbenvincent.github.io/delay-discounting-analysis/) ^60,69^. This approach has the benefit of increasing the reliability of parameter estimates by estimating group parameter distributions to constrain the estimates of individual discounting parameters. Participants’ subjective values (*SV*) of each of the two options were modeled using the hyperbolic discount function. This function discounts the value of delayed rewards according to the equation:

$$SV=\frac{A}{1+kt}$$

where A is the reward amount, t is the time to the reward and k is the discount rate. The toolbox generates a natural log-transformed discount rate (log(k)) for each participant, which normalizes the distribution of discount rates. Decisions were modeled using the default function in the package, which models decisions as a noisy function of the difference in subjective values between the two options:

$$p(\mathrm{Choose}\;LL)=\varepsilon +(1-2*\varepsilon )\varphi \left(\frac{SV_{LL}-SV_{SS}}{\alpha }\right)$$

where φ() is the cumulative normal function, ε is a noise parameter governing the likelihood of random responding (higher ε indicates more random choices), and α is a noise parameter governing the acuity of the value comparison (as higher α values reflect reduced sensitivity to the subjective value difference between the options).

We fit low and high-risk groups separately to maintain statistical independence between the hierarchically fitted parameters for the two groups. Through piloting, we designed our choice set to sample the range of discount rates observed in typical subject samples (with unknown risk of anorexia). We incorporated the prior expectation that our choice set would evenly sample discount rates into the model by setting the prior on the group log(k) to a value that would predict an approximately equal number of larger-later and smaller-sooner preferences in our choice set. We used the package default for the variance of the prior, resulting in a *N*(−5.3, 2.5) prior on log(k). Using Markov Chain Monte Carlo, we ran four independent sampling chains for 25,000 samples each and discarded the first 1,000 samples of each chain as burn-in, resulting in 96,000 samples from the posterior distribution. To assess whether the sampling procedure converged to the posterior distribution, we computed the $\overset{ˆ}{R}$ statistic, a measure of whether the four chains converged to the same distribution, for all parameters. No participants’ parameter values had $\overset{ˆ}{R}>1.01$, indicating chain convergence. The fitted model explains participant behavior well, correctly classifying 86.8% of choices. The mean and standard deviation of the key parameters of the model are reported in *Supplementary Table 4*.

Detailed Methods and Results for the Ebert-Prelec model are detailed in Supplemental Material (*Supplementary Methods* and *Supplementary Results*)

##### Primary Analyses.

Differences in discount rates [log(k)] and future orientation by anorexia risk group (low, high) were examined using independent-samples t-tests as the primary analysis following our preregistered analysis plan, given the established clinical significance of the EAT cutoff value adopted here and elsewhere ^24,25^. Following our pre-registration and in alignment with the RDoc framework, we additionally examined linear associations amongst those variables using a continuous approach as additional analysis using Pearson’s correlation coefficients (see *Supplementary Results*).

Following our pre-registered analysis plan, one-sided hypotheses regarding the association between anorexia risk, intertemporal discounting, and future orientation (https://osf.io/h46x2) were tested using one-tailed p-values. Two-tailed p-values were used for analysis pre-registered as exploratory (temporal processing tasks) and/or for any analysis that were not originally pre-registered—such as dispositional negativity and the mediation analysis between risk group, future orientation, and intertemporal discounting (see *Supplementary Materials* for a complete description of any additional analyses that were conducted beyond the pre-registered plan).

##### Exploratory Analyses

We tested whether time estimation accuracy (Volle task and Zimbardo task scores) varied by anorexia risk using independent sample t-tests. We examined whether anorexia risk (EAT-26 scores) correlated with temporal accuracy continuously using Pearson’s correlation. As mentioned, all exploratory analysis were conducted using two-tailed *p* values.

Given the comorbidity of anorexia and anxiety disorders, we examined whether Dispositional Negativity varied by risk group, and entered Dispositional Negativity as a covariate in control analysis to test whether future-oriented cognition explained anorexia risk after controlling for dispositional negativity scores.

Finally, because we observed the hypothesized group differences on intertemporal choice and future-oriented cognition as a function of anorexia risk, and in light of prior work suggesting prospection and future-oriented cognition as an important mechanism underlying intertemporal choice, we conducted an exploratory mediation analysis to determine whether future-orientated cognition explained (i.e., statistically mediated) anorexia risk group differences in intertemporal discounting. To do so, we used ordinary least squares regression via the PROCESS software version 4.3.1 in R (Hayes, 2022). Evidence for mediation was obtained using a bootstrap confidence interval (n=10,000 samples) for the partial standardized indirect effect ^70^.

##### Control analysis: gender and income

We examined the possible confounding influence of gender and income in intertemporal discounting behavior, anorexia risk, and future-orientation (using log(k), EAT-26 scores, and future-orientation factor scores, respectively). We tested for gender differences using independent sample t-tests. We recorded participants’ monthly disposable incomes according to the scale (1: 0-$500, 2: $500-$1000, 3: $1000-$1500, 4: $1500-$2000, 5: $2000-$2500, 6: $2500-$3000, 7: $3000+), and examined the impact of income differences on delay discounting, anorexia risk, and future-orientation using a one-way ANOVA.

## Results

### Individuals at risk for anorexia are less impulsive, showing reduced intertemporal discounting

**Figure 2.**
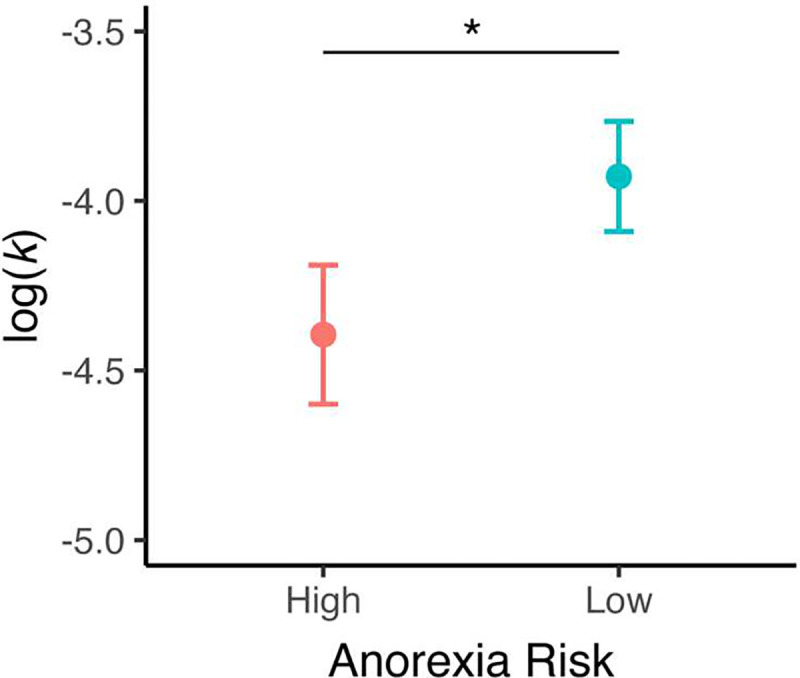
Anorexia risk and intertemporal decision making. Individuals at higher risk for anorexia show reduced delay discounting compared to a low-risk group. *p < 0.05

Individuals in the anorexia risk group devalued future rewards *less steeply* than individuals in the low-risk group, as indicated by significantly lower intertemporal discounting rates in anorexia-high vs. low-risk groups (log(k) M_High-risk_= −4.394, SD_High-risk_ = 1.434 vs. M_low-risk_= −3.928, SD_low-risk_ = 1.514, *t*_(134)_= −1.758, *p* = 0.041, dz = 0.314) (Figure 2). This result extends prior insights to a non-clinical, never-diagnosed sample and suggests that reduced intertemporal discounting might be a pre-existing, risk factor for anorexia.

### Individuals at risk for anorexia show increased future-oriented cognition

**Figure 3.**
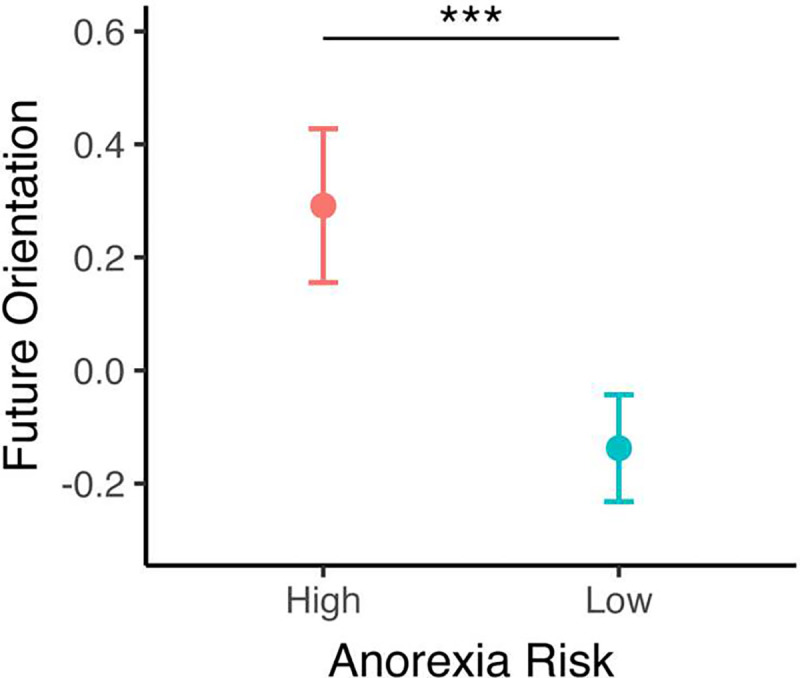
Anorexia risk and temporal orientation. Anorexia risk is associated with a future-oriented cognitive style. ***p < 0.005

Next, we examined whether a putative cognitive mechanism underlying intertemporal discounting—the propensity to engage in prospective, future oriented thinking during everyday decisions—might further characterize anorexia risk and explain anorexia-risk group differences in intertemporal choice behavior. First, in agreement with prior work ^18–21^, intertemporal discounting rates and future-orientation factor scores were negatively associated across the sample, such that higher future-oriented cognition predicted lower intertemporal discounting [log(k)] (*r*_(132)_=−0.26, Pearson’s *p* = 0.002). Critically, we found a significant group difference in future oriented cognition between groups, whereby individuals in the anorexia risk group had significantly higher future-orientation factor scores compared to the low-risk group (M_high-risk_= 0.292, SD_high-risk_ = 1.007 vs. M_low-risk_= −0.138, SD_low-risk_ = 0.916, *t*_(147)_= 2.659, *p* = 0.004, dz = 0.451) (Figure 3). This novel result extends a prior finding obtained from a clinical sample to a non-clinical, risk population, and indicates that future-oriented cognition might be a mechanism that drives intertemporal discounting differences between anorexia risk groups.

### Future-oriented cognition mediates the association between anorexia risk and intertemporal decision-making

**Figure 4:**
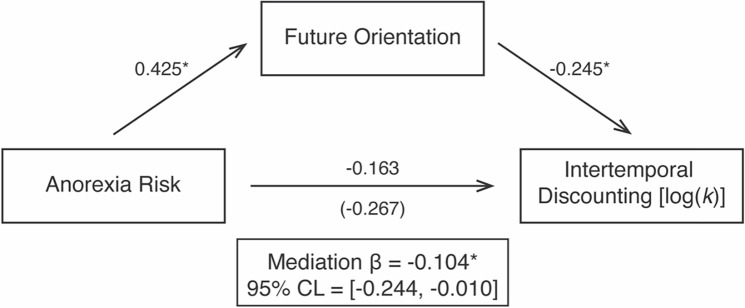
Future oriented cognition significantly mediated the association between anorexia risk and intertemporal discounting. Mediation paths with standardized regression coefficients for each path are shown. The total effect relating anorexia group and intertemporal discounting rate is shown in parentheses. * *p* < 0.05.

In light of prior work suggesting that prospective, episodic future thinking may be an important cognitive factor that underlies intertemporal behavioral choices, we examined whether the anorexia risk group difference we observed in intertemporal discounting was accounted for by differences in future oriented cognition between groups. To that end, we conducted a mediation analysis (see Methods). As detailed in Figure 4, individuals in the anorexia high-risk group showed higher future-orientation scores than the anorexia low-risk group (ß = 0.425, SE = 0.174, t = 2.389, *p* = 0.018). Critically, a future-oriented cognitive style predicted *less* intertemporal discounting behavior [log(k)] (ß = −0.245, SE = 0.131, t = −2.852, *p* = 0.005). A bootstrap analysis of the partial standardized indirect effect (ß= −0.104, SE = 0.061) showed a significant mediation [95% CI = (−0.244, −0.010)], such that future orientation mediated group differences in anorexia risk. Accordingly, after controlling for future orientation scores, we found that the association between anorexia group and log(k) values was reduced (total effect: ß =−0.267, SE = 0.267, t = −1.482, *p* = 0.141, partial effect: ß = −0.163, SE = 0.266, t=−0.909, *p* = 0.365). In sum, these results provide novel evidence that a future-oriented cognitive style underlies behavioral differences in intertemporal decision making in individuals at risk for anorexia.

### No reliable differences in ‘subjective’ time perception by anorexia risk

To test whether anorexia risk modulated subjective time perception, we examined the performance of individuals at high vs. low risk for anorexia in two previously-validated temporal processing tasks: the Volle task and the Zauberman task (see Methods). We found no reliable group differences in performance for either task (Zauberman: M_High-risk_= −0.324, SD_High-risk_ = 1.77 vs. M_low-risk_= 1.172, SD_low-risk_ = 11.963, *t*_(142)_= −0.904, *p* = 0.368, d_z_ = 0.156) nor correlations with anorexia-risk severity (Volle 1s interval and EAT-26: *r*_(148)_=0.049, *p* = 0.549; Volle 2s interval and EAT-26: *r*_(148)_=0.023, *p* = 0.781; Zauberman and EAT-26: *r*_(142)_=−0.058, *p* = 0.49).

### Controlling for mood and anxiety

As expected, participants at risk for anorexia had higher negative mood and anxiety symptoms, as reflected by dispositional negativity scores, compared to the low-risk group (*t*_(148)_=4.088, *p* < 0.001, d_z_= 0.69) (*Supplementary Figure 2*). Accordingly, EAT-26 scores and dispositional negativity were positively correlated (*r*_(148)_ = 0.314, *p* < 0.001; *Supplementary Figure 3).* In light of this group difference and potentially confounding factor between groups, we next examined whether dispositional negativity was associated with intertemporal discounting and future orientation in our sample. Dispositional negativity was unrelated to intertemporal choice in the current sample (*r*_(132)_= 0.052, *p* = 0.551). Interestingly, dispositional negativity was *negatively* associated with future-orientation scores (*r*_(147)_= −0.290, *p* < 0.001), and these two factors each accounted for independent variance in anorexia risk, as follows. When entered in a simultaneous regression model predicting anorexia risk, future orientation and dispositional negativity were both significant and independent predictors of EAT scores (B_Future-orientation_ = 3.825, *SE* = 1.026, *t* = 3.729, *p* < 0.001; B_Dispositional Negativity_ = 4.973, *SE* = 1.004, *t* = 4.954, *p* < 0.001) (*Supplementary Figure 4*). Collectively, these analyses suggest that future-oriented cognition, and mood and anxiety symptomatology, while both linearly associated with severity of anorexia symptoms, *independently* explained anorexia risk. Moreover, only future-oriented cognition, and not dispositional negativity, was associated with intertemporal choice in this never-diagnosed sample of individuals at risk for anorexia.

### Controlling for gender and income

Gender and income themselves might influence intertemporal discounting behavior, anorexia risk and future-oriented cognition. Therefore, we next examined whether gender and income differed as a function of each of these factors. First, we did not find that gender and income modulated delay discounting, future oriented cognition, and/or that they were differentially associated with anorexia risk in our sample Gender: log(k): *t*_(131)_ = −0.476, *p* = 0.635; future-orientation: *t*_(144)_ = 0.207, *p* = 0.837; EAT-26: *t*_(147)_ = −0.776, *p* = 0.439; Income: log(k): *F*_(6, 99)_ = 0.743, *p* = 0.616; future-orientation: *F*_(6, 109)_ = 1.324, *p* = 0.253; EAT-26: *F*_(6, 112)_ = 1.211, *p* = 0.306).

Nonetheless, we next examined whether anorexia risk continued to predict intertemporal discounting *after* controlling for gender and income. Anorexia risk continued to predict reduced delay discounting (*F*_(1, 97)_= 5.070, *p* = 0.001) and greater future-orientated cognition (*F*_(1, 107)_= 9.532, *p* = 0.003) after entering gender and income as covariates in an ANOVA model. Next, we examined whether future-orientation continued to mediate the association between anorexia risk and delay discounting behavior after controlling for gender and income. To that end, we ran a parallel mediation model by entering future-orientation factor score, gender and income as parallel mediators in the same model. We found that a future-oriented cognitive style continued to predict reduced intertemporal discounting log(k) (ß = −0.285, SE = 0.150, t = −2.883, *p* = 0.005), but not gender (ß = 0.003, SE = 0.292, t = 0.026, *p* = 0.98) or income (ß = 0.077, SE = 0.085, t = 0.813, *p* = 0.418). A bootstrap analysis of the partial standardized indirect effect (ß = −0.166, SE = 0.077) provided evidence for a significant mediation [95% CI = (−0.334, −0.038)], whereby future orientation continued to mediate group differences in anorexia risk. In contrast, neither gender [ß= 0.001, SE = 0.053, 95% CI = (−0.100, 0.12)] nor income [ß= 0.005, SE = 0.03, 95% CI = (−0.300, 0.091)] were significant mediators of the association between anorexia risk and intertemporal discounting. After controlling for future orientation scores, gender, and income, the association between anorexia group difference and log(k) values went from significant to non-significant (total effect: ß =−0.394, SE = 0.298, t = −1.989, *p* = 0.049, partial effect: ß = −0.234, SE = 0.312, t=−1.131, p = 0.261), suggesting a full mediation effect ^70^. In summary, the above-reported findings, which suggest that anorexia risk is linked to reduced intertemporal discounting via increased future orientation, fully replicated after controlling for gender and income.

### Further ascertaining the sensitivity of delay discounting to anorexia (vs. bulimia) risk

As mentioned above, the present study assessed risk for anorexia using the Eating Attitudes Test (EAT-26), which was originally validated using a sample of anorexia nervosa patients ^24^. However, although the EAT-26 has excellent sensitivity and specificity for DSM-IV eating disorders diagnoses ^25^, it was not designed to discriminate between anorexia versus bulimia nervosa. As stated previously, we referred to risk as indexed by this questionnaire as ‘anorexia risk’ given its historical origin, in addition to the recognition that anorexia and bulimia may exist in a continuum ^26–28^. However, anorexia and bulimia have been differentially linked to *reduced* versus *increased* delay discounting, respectively ^2,4^. Therefore, even though the current study and the EAT-26 questionnaire are not ideally suited to differentiate between anorexia vs. bulimia diagnostic categories, given the known differential association between them and delay discounting, we re-analyzed our data after excluding EAT-26 items that measure binge-eating and purging behavior (items 4, 9, and 25). Then, we recomputed high- and low-risk groups as previously described (Methods) to examine whether anorexia risk—now estimated after the exclusion of bulimia-related symptoms—continued to predict reduced intertemporal discounting and increased future orientation in this sample. We fully replicated our results, as follows: anorexia risk continued to predict lower intertemporal discounting (M_High-risk_= −4.495, SD_High-risk_ = 1.381 vs. M_low-risk_= −3.905, SD_low-risk_ = 1.520, *t*_(134)_= −2.182, *p* = 0.015, dz = 0.4) and higher future-oriented cognition (MHigh-risk= 0.314, SDHigh-risk = 1.005 vs. Mlow-risk= −0.127, SDlow-risk = 0.922, *t*(147)= 2.674, *p* = 0.004, dz = 0.464). Moreover, future-oriented cognition continued to mediate the association between anorexia risk and intertemporal discounting [ß= −0.105, SE = 0.063, 95% CI = (−0.253, −0.010)].

## Discussion

Eating disorders, including anorexia, are highly prevalent, challenging to treat, and associated with the highest mortality rate of all psychiatric conditions ^71^. Yet, the neurocognitive mechanisms that drive the persistent overriding of food in the service of long-term goals in anorexia remain unclear. Here, we replicated and extended previous findings indicating reduced intertemporal discounting in acute anorexia to a sample of at-risk but never diagnosed individuals. Moreover, our results specified a cognitive mechanism that may underlie the propensity of individuals with anorexia to value delayed (vs. present-moment) rewards: temporal orientation, whereby anorexia risk was robustly associated with a future-oriented cognitive style. These results were unrelated to subjective time perception and held independently of mood and anxiety symptomatology differences between groups. In summary, our study shows that a future-oriented cognitive style robustly characterizes individuals at risk for anorexia and may be a mechanism underlying reduced intertemporal discounting behavior in this population.

The propensity to prefer larger future rewards—as opposed to smaller-but-sooner rewards—is thought to depend on distinct yet mutually interactive systems: reward valuation, cognitive control, and prospective systems ^2,3,29,66,72,73^. The prospective system, while studied less frequently, interfaces with the valuation system with the potential to reduce myopic decision making, therefore promoting decisions that are consistent with increased cognitive control. Specifically, engaging in episodic future thinking is associated with reduced delay discounting and a shift *away* from hedonic choices in health domains, such as smoking and drinking behavior ^49,50^ (reviewed in ^2^). Put differently, engaging the prospective system, which relies on extended memory regions including the hippocampus, precuneus, and frontal pole, is thought to facilitate future-minded decision making ^3,74,75^. Accordingly, in our study, future oriented cognition correlated negatively with delay discounting across individuals. However, whether future-oriented cognition contributes to the previously reported ‘imbalance’ of self-control and hedonic reward valuation systems in anorexia ^8,9^—and explains reduced delay discounting in this population ^4^—had not previously been tested. Here, we show a robust difference in future-oriented cognition in individuals at risk for anorexia (compared to a low-risk group). Further corroborating the relevance of this phenotype for eating disorder risk, we found that across individuals, anorexia risk (EAT-26 questionnaire scores) correlated positively with future-oriented cognition. Moreover, future oriented cognition mediated the reduced intertemporal discounting we observed in individuals at risk for anorexia. Collectively, these results suggest that larger/delayed (vs. smaller/present-moment) rewards may be valued more by individuals at risk for anorexia in part due to the propensity of those individuals to spontaneously consider the future during their everyday life decisions. Overall, these insights align with prior accounts suggesting an imbalance of reward valuation vs. control systems in anorexia—and offer a unified cognitive mechanism that may underlie precisely such imbalance.

These results have implications for our understanding of eating disorders symptom and illness trajectories. Delay discounting findings in subclinical and acute anorexia indicate that future rewards hold a higher value among those at-risk of developing anorexia than for healthy controls. This is markedly different from what is seen in other psychiatric illnesses in which there are typically abnormally low levels of self-control, as indexed by higher intertemporal discounting, such as in substance abuse disorders, illustrating that the intertemporal discounting construct may be a useful indication of maladaptive cognitive patterns at both ends of the spectrum ^2,4,39^. In particular, the increased ability to delay rewards in anorexia may contribute to disordered behaviors such as persistent dieting and food restriction via the overvaluation of future goals. It is possible that individuals who habitually engage in future thinking view future rewards as more concrete, which has been shown to increase the relative value of future rewards, and be associated with less discounting ^47,51^. As alluded to above, the ability to vividly imagine potential future consequences and outcomes of our actions has been suggested to be a proximal mechanism by which prospective thinking promotes future-oriented decision making and facilitates cognitive control over actions in the present ^5,76^. Therefore, future work should examine whether imagery of future outcomes may be more vivid and/or concrete in anorexia. Relatedly, results from a recent study suggest that increased engagement of extended memory neural systems during the viewing of thinness-related pictures predicts the persistence of eating pathology ^77^. Thus, future work could also examine whether prospective memory systems are more functionally connected with valuation and cognitive control systems in anorexia compared to healthy controls, which may underlie increased future-oriented thinking and decision making in this population.

The finding that individuals at risk for anorexia show reduced delay discounting compared to a low-risk group extends prior findings in acute anorexia to a subclinical, never-diagnosed sample. This result, combined with the robust difference in future-oriented cognition observed between anorexia risk groups, converge to suggest that altered future-oriented cognition *and* decision making may constitute an endophenotype (or vulnerability factor) for anorexia. However, while these findings are consistent with a meta-analysis of delay discounting in anorexia ^2,4^, a prior longitudinal study did not find altered delayed discounting in anorexia following treatment and remission ^15^ (for related null findings in remitted anorexia, see ^17,40,41^). If intertemporal discounting is an endophenotype of anorexia, reduced intertemporal should persist beyond treatment. It is possible that current interventions for anorexia—such as Cognitive Behavioral Therapy ^78^, or the Body Project Treatment ^79,80^—may target processes that overlap with the mechanisms that drive intertemporal choice, for instance, by redirecting patients’ primary goals away from weight and control related to other domains ^78^, and/or by helping participants ‘devalue’ long-term, thin ideals ^79^. Future work tracking individuals at risk for anorexia prospectively and longitudinally—with repeated measurements of future oriented cognition *and* intertemporal discounting behavior—will be required to fully ascertain whether the hereby reported cognitive and behavioral alterations are merely correlates of anorexia risk, or whether they may indeed constitute a reliable vulnerability factor that could be identified by clinicians and proactively targeted via cognitive interventions prior to anorexia onset ^81^. For instance, interventions that alter construal level (from more concrete to more abstract) have been shown to modulate the value ascribed to proximal vs. distal rewards ^82^—and would therefore be promising future avenues for anorexia treatment.

In light of prior work suggesting a role for subjective time perception in delay discounting and impulsive decision making ^44,45,55–58^, we included assessments of time perception in our study, which were examined in relation to anorexia risk in pre-registered exploratory analyses. Contrary to our hypotheses, we did not find that time was experienced faster (and/or more accurately) in individuals at risk for anorexia. This contrasts with a previous report in acute anorexia, which showed accelerated time estimates in clinically diagnosed individuals with anorexia compared to controls ^59^. Collectively, these findings suggest that while temporal factors do play an underappreciated and seldom-studied and role in anorexia, it is the *valuation* of rewards and consequences across time, into the future, that characterizes anorexia vulnerability—rather than a change in one’s internal clock ^45^.

The following limitations from the current study warrant further investigation. First, the data for our study were collected online (during the COVID-19 pandemic). Given the inherent challenges associated with providing adequate participant monitoring and timely psychological assistance, if necessary, in the online data collection environment, we removed what we considered sensitive questions from our study for ethical reasons—including questions about participants’ BMI. Therefore, although we refer to our sample as ‘subclinical’ given that they self-reported not having been previously diagnosed with an eating disorder, it is possible that the anorexia risk group included individuals who might meet clinical diagnostic criteria for anorexia, which we would have been able to better assess had their BMI data been collected and/or a formal clinical diagnostic interview conducted. Relatedly, in the absence of BMI information, we were unable to fully differentiate between anorexia versus bulimia risk with the EAT-26 questionnaire—although note that analyses excluding bulimia-related items from the final EAT-26 scores (and group risk assignment) replicated our originally reported results. In the clinic, these disorders have been shown to be differentially liked to intertemporal discounting—with acute anorexia predicting *reduced*, and bulimia predicting *increased*, intertemporal discounting behavior compared to healthy controls ^4,39^. Therefore, future work should acquire BMI data to unambiguously ascertain the (sub)clinical status of at-risk individuals, and to further disambiguate whether anorexia and bulimia risk are differentially linked with intertemporal discounting behavior.

In closing, our results demonstrate that never-diagnosed individuals at risk for anorexia are *willing to wait*, showing future-oriented cognition and decision making compared to a low-risk group. Together, these findings pave the way for future longitudinal work with at-risk samples to fully elucidate the import of the delay discounting as an endophenotype for anorexia and determine the role of the prospective neural network systems for anorexia vulnerability.

## Data Availability

Here, we report how we determined our sample size, all data exclusions (if any), all manipulations, and all measures in the study. The study follows JARS (Appelbaum, et al., 2018). This study was preregistered on OSF (https://osf.io/h46x2). All data, analysis code, and research materials are available at: https://osf.io/3y7dq/.

## Footnotes

1 As detailed in the Methods, the present study assessed anorexia/eating disorders risk using the Eating Attitudes Test (EAT-26), which was originally validated using a sample of Anorexia Nervosa patients ^24^. The EAT-26 has excellent sensitivity and specificity for DSM-IV eating disorders diagnoses ^25^—however, this questionnaire was not designed to discriminate between Anorexia and Bulimia Nervosa. Therefore, while we refer to eating disorders risk as ‘Anorexia risk’ given the historical origin of the questionnaire, in addition to the growing recognition that Anorexia and Bulimia may exist in a continuum ^26–28^, the current study does discriminate between Anorexia (e.g., restrictive vs purging) subtypes, or between Anorexia and Bulimia; but see “Further ascertaining the sensitivity of delay discounting metrics anorexia risk” for a re-analysis of the data excluding bulimia-related EAT-26 items prior to risk group assignment.
